# Locating human splenic capillary sheaths in virtual reality

**DOI:** 10.1038/s41598-018-34105-3

**Published:** 2018-10-24

**Authors:** B. S. Steiniger, V. Wilhelmi, M. Berthold, M. Guthe, O. Lobachev

**Affiliations:** 1 Institute of Anatomy and Cell Biology, University of Marburg, Marburg, D-35037 Germany; 2 Visual Computing, Institute of Computer Science, University of Bayreuth, Bayreuth, D- 95440 Germany

## Abstract

Stromal capillary sheath cells in human spleens strongly express CD271, the low affinity nerve growth factor receptor p75. Serial sections of a representative adult human spleen were double-stained for CD271 versus smooth muscle alpha actin (SMA) plus CD34 to visualise capillary sheaths, the arterial tree and endothelial cells by transmitted light. Preliminary three-dimensional (3D) reconstructions of single regions were inspected in virtual reality (VR). This method showed that a large number of CD271^+^ sheaths occur in a post-arteriolar position often surrounding capillaries located close to divisions of arterioles. The length and diameter of capillary sheaths are rather heterogeneous. Long sheaths were observed to accompany one or two generations of capillary branches. We hypothesise that human splenic capillary sheaths may attract recirculating B-lymphocytes from the open circulation of the red pulp to start their migration into white pulp follicles along branches of the arterial tree. In addition, they may provide sites of interaction among sheath macrophages and B-lymphocytes. Our innovative approach allows stringent quality control by inserting the original immunostained serial sections into the 3D model for viewing and annotation in VR. Longer series of sections will allow to unequivocally localise most of the capillary sheaths in a given volume.

## Introduction

The existence of capillary sheaths in human spleens and the first inter-species comparison of these structures have been reported about 150 years ago. Schweigger-Seidel^1^ described the location of the sheaths around post-arteriolar capillaries and published an almost correct drawing of a longitudinally sectioned sheath. Solnitzky^2^ confirmed this location and regarded the sheaths as macrophage accumulations, an opinion that was shared by several subsequent authors. Attempts at reconstructing human capillary sheaths from serial sections exist^3^. However, the exact shape of the sheaths, their arrangement in the vasculature, their cellular composition and their function have remained enigmatic.

Our recent studies showed that human splenic capillary sheaths consist of three main cell types surrounding capillary endothelia, namely CD271^+^ stromal capillary sheath cells, CD68^+^CD163^−^ macrophages and recirculating B-lymphocytes^4,5^. Up to now, most descriptions of human capillary sheaths have missed one of these cell types. Human capillary sheaths are morphologically similar to those of other species including birds, the sheaths of which have been described in most detail^6–11^. The term “ellipsoid” is commonly used to describe non-human capillary sheaths, but this term does not seem to be morphologically adequate for any species investigated so far^4,12,13^. It may be derived from misinterpretations of splenic white pulp compartments in the early days of histology. Capillary sheaths do not exhibit an elliptical shape, but they are elongated structures accompanying capillary branches of first, second or even higher order^3,6,12,13^. In birds, capillary sheaths consist of stromal cells, macrophages and a thick peri-ellipsoidal B-cell sheath. This sheath represents a distinct B-cell compartment in addition to follicles^6,14,15^ and is dependent on the existence of the bursa^16^. One of the cell types in avian and other vertebrate capillary sheaths - most probably macrophages - is responsible for uptake of immune complexes and particulate materials from the blood^7,17,18^. This is also true for fish ellipsoids^19,20^.

It has been known for a long time, that mouse and rat spleens do not possess capillary sheaths. Apparently, rodents and lagomorphs^2^ do not exhibit these microanatomical structures in their spleens. In consequence, the dominance of mouse-based research has led to a more or less total neglect of splenic capillary sheaths.

We now present a first and preliminary step towards analysing the three-dimensional arrangement of human splenic capillary sheaths. This leads to a hypothesis on their function in the context of the open circulatory system of the splenic red pulp.

## Results

The distribution of CD34, SMA and CD271 in human spleens have been described in previous publications^4,5,21,22^. Briefly, CD34 occurs in endothelia of capillaries and large vessels, in adventitial fibroblasts of large vessels and in superficial fibroblasts of trabeculae. In addition, perifollicular sinus endothelia are faintly stained. SMA is present in vascular smooth muscle cells, in perivascular fibroblasts and in marginal reticular cells surrounding the splenic white pulp, especially the follicles. Subcapsular and trabecular fibroblasts express SMA. Ubiquitous fibroblasts in the red pulp cords are faintly positive. CD271 is also weakly present in ubiquitous red pulp fibroblasts, in vascular adventitial fibroblasts and in the fibroblastic reticulum cells (FRCs) of T-lymphocyte zones. The most strongly CD271-positive cells are the more or less isoprismatic stromal cells of capillary sheaths. Follicular dendritic cells (FDCs) are also rather strongly stained.

**Figure 1:**
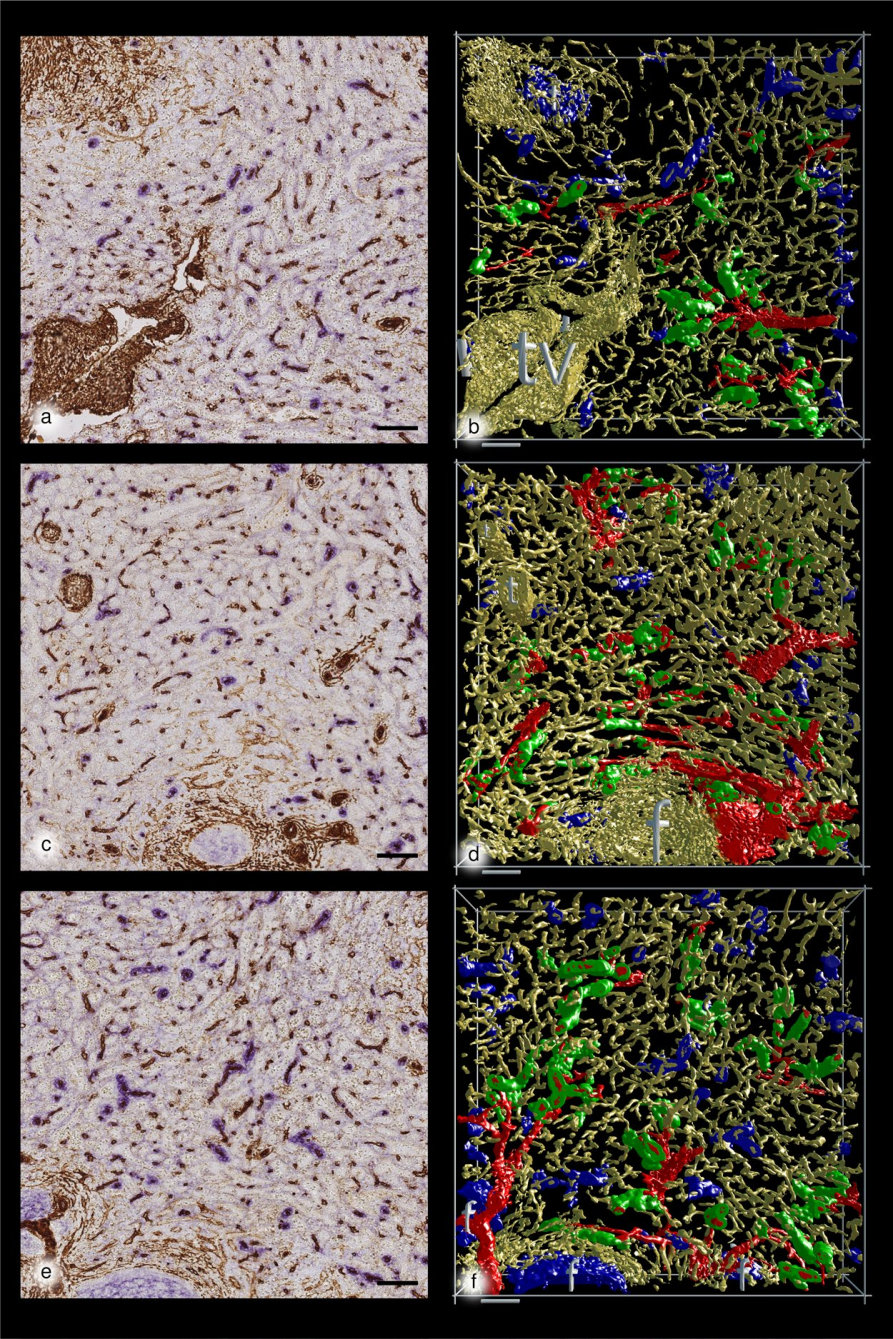
Immunostained first sections and 3D models of R1 to R3 showing the location of capillary sheaths First section of R1 (**a**), R2 (**c**) and R3 (**e**) stained for CD34 plus SMA in brown and CD271 in blue. The first sections are compared to the 3D-model of R1 (**b**), R2 (**d**) and R3 (**f**) showing staining for CD34 plus SMA in yellow, for post-arteriolar CD271^+^ capillary sheaths in green and for capillary sheaths of undetectable location in blue. CD271^+^ FDCs in follicles are also blue. The direct connections of arterial vessels to green sheaths were manually marked in red. The iso-values were chosen to exclude weakly CD34^+^ perifollicular sinus endothelia and weakly CD271^+^ interstitial fibroblasts without compromising microvesssel continuity. As a consequence of this, the diameter of microvessels differs among the ROIs. Scale bars = 100 µm, f = follicle, t = trabecula, tv = trabecular vein.

**Figure 2:**
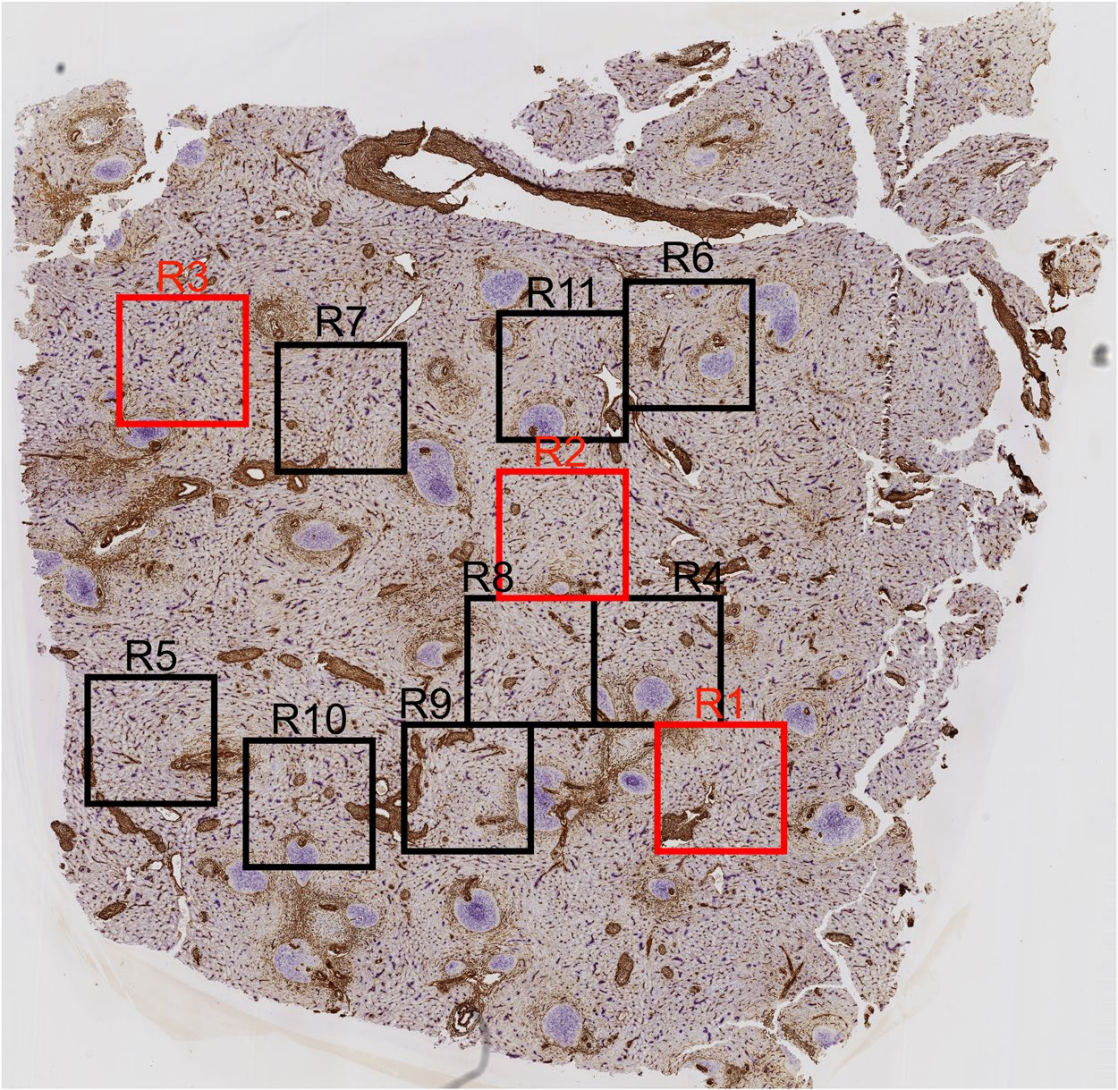
Overview of all 11 regions (R) visualised in 3D.

A paraffin-embedded sample of a normal human spleen which had been studied previously^23^ and which was found representative of the majority of adult human spleens with small symmetric secondary follicles was processed to 24 serial sections which were simultaneously stained for CD34 and SMA in brown colour and for CD271 in blue (Fig. 1a,c,e). The sections were scanned and 11 regions of interest (ROIs) each representing a volume of 0.17 mm^3^ were selected for study (Fig. 2). The ROIs were chosen to always contain red pulp tissue, but it was inevitable that parts of follicles, trabeculae and larger arteries or veins were also included.

**Figure 3:**
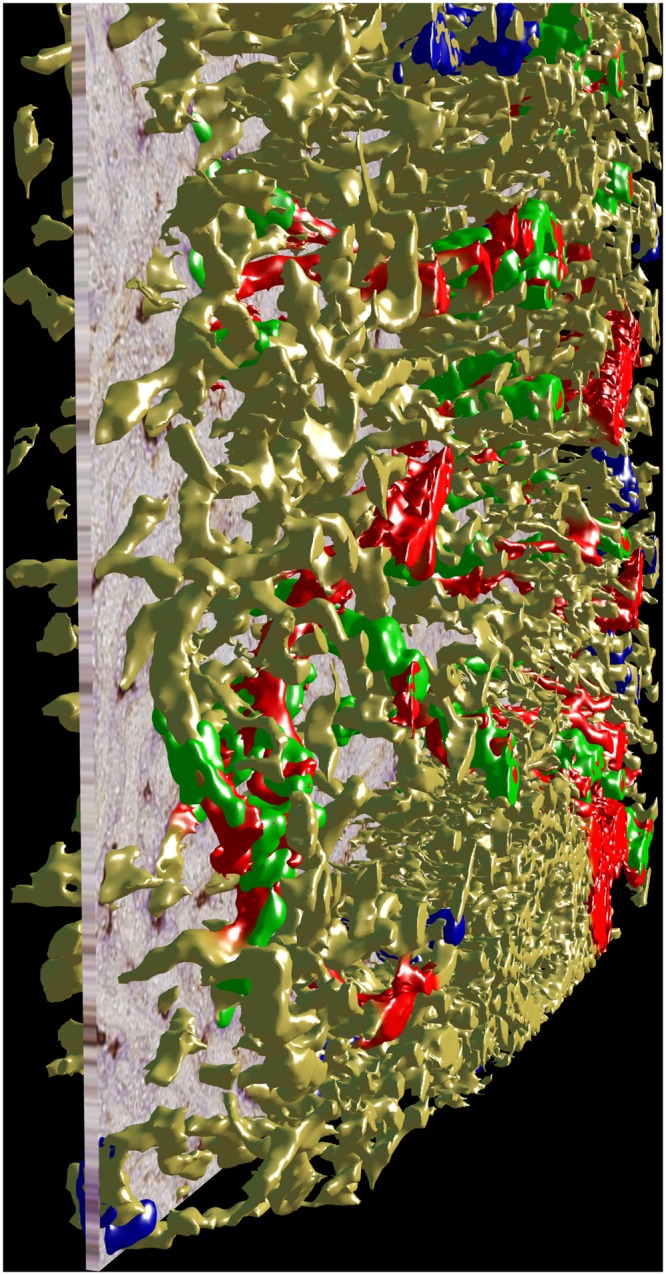
A serial section blended into the 3D model of R2 showing staining for CD34 plus SMA in yellow, for post-arteriolar CD271^+^ capillary sheaths in green and for capillary sheaths of undetectable location in blue. The direct connections of arterial vessels to green sheaths were manually marked in red.

After appropriate processing, 3D models representing CD34 plus SMA in yellow and CD271^+^ stromal capillary sheath cells in blue or green colour were generated and inspected in virtual reality (VR), (Figs 1b,d,f and 3; Supplementary Videos S1–3: https://zenodo.org/record/1229434; Supplementary Files S1–11: 10.5281/zenodo.1229434). In detail, capillary sheaths were detected automatically by using a threshold on the cyan colour channel. By this method, all voxels brighter than a certain intensity were regarded as constituting sheaths. In addition, FDCs in follicles were detected. Each original immunostained section could be blended into the VR model to control the quality of the reconstruction (Fig. 3; Supplementary Video S4: https://zenodo.org/record/1229434/files/Video_S4.mov; Supplementary Files S1–11: 10.5281/zenodo.1229434). Arterial vessels were defined by the presence of a layer of SMA^+^ smooth muscle cells surrounded by branched adventitial SMA^+^ fibroblasts and could thus be distinguished from capillaries lacking such a surrounding. In order to alleviate recognition, the surface of those arteries and arterioles (partially including their adventitial cells) which led to capillary sheaths were manually annotated in the model and coloured red using mesh colouring with geodesic distances in VR. In the next step, all CD271^+^ capillary sheaths, which were contacted by SMA-positive arterioles were manually attributed a green colour, irrespective of the fact whether they were completely or only partially contained in the model. All other sheaths which could not be unequivocally related to a feeding arteriole were visualised in blue colour. Thus, blue sheaths might also be located in a post-arteriolar position, but this could not be diagnosed, because the respective sheath or its adjacent feeding vessel had been cut at the surface of the model.

**Figure 4:**
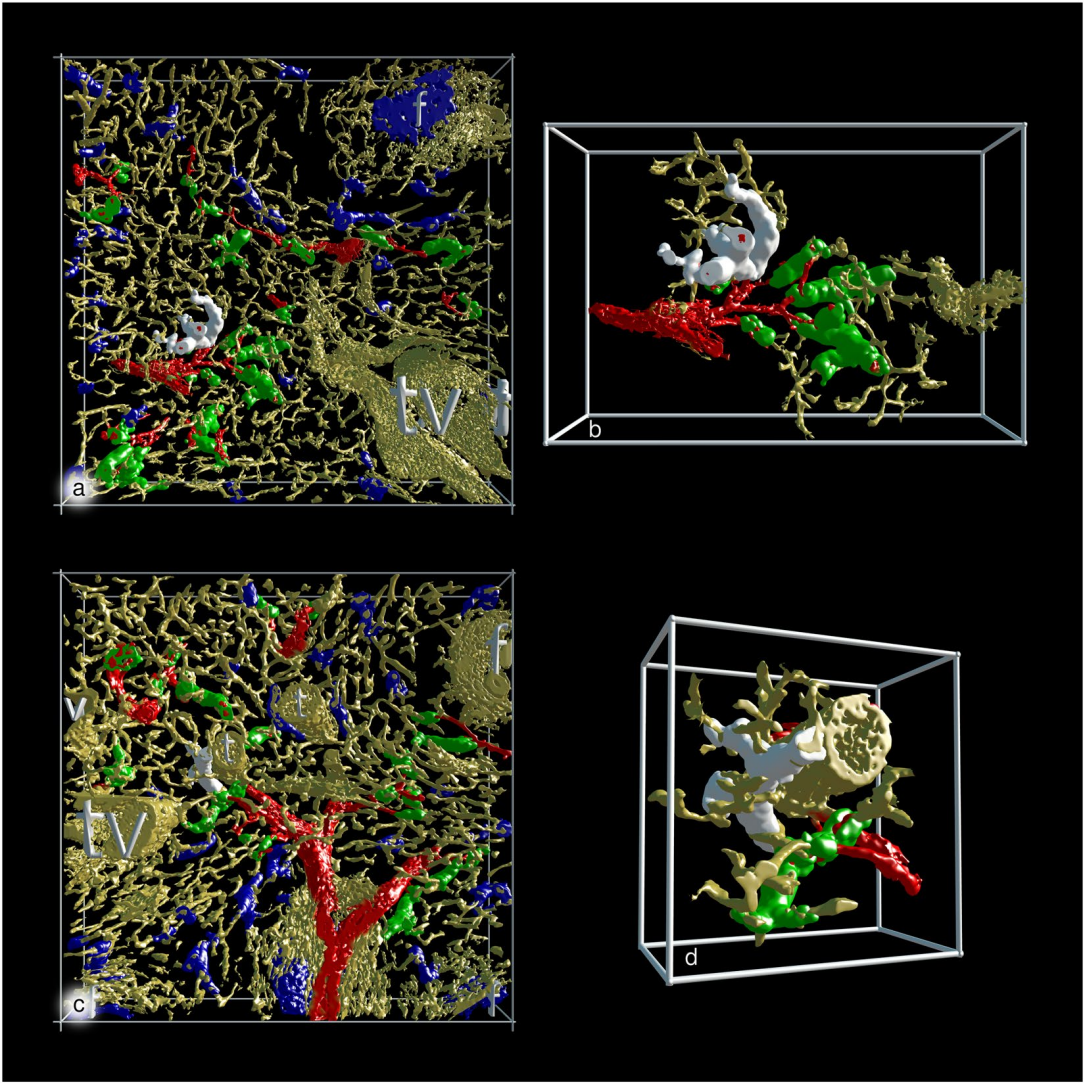
3D visualisation of the two largest sheaths found in all regions investigated. (**a**) The largest sheath with a demonstrable connection to an artery highlighted in white colour in R1 (view from last section of the series). (**b**) Same sheath as in (**a**) at higher magnification and after removal of non-connected structures. (**c**) The largest sheath without demonstrable connection to an artery highlighted in white colour in R10 (view from last section of the series). (**d**) Same sheath as in (**c**) at higher magnification and after removal of non-connected structures. (**a**–**d**) show staining for CD34 plus SMA in yellow, for post-arteriolar CD271^+^ capillary sheaths in green and for capillary sheaths of undetectable location in blue. In (**a**) and (**c**) CD271^+^ FDCs in a follicle are also blue. The direct connections of arterial vessels to green sheaths were manually marked in red. Length of the horizontal part of the bounding box = 1 mm in (**a**) and (**c**); 596 µm in (**b**) and 293 µm in (**d**). f = follicle, t = trabecula, tv = trabecular vein, v = vein.

By this method, we found that capillary sheaths were much longer than anticipated. Most of them were not completely contained in the section series covering about 150 µm. Altogether, we found 528 capillary sheaths in the 11 ROIs, i.e., in a volume of 1.85 mm^3^ (Supplementary Table S1: https://zenodo.org/record/1229434/files/table_S1.xlsx). 44,5% of all sheaths were observed in a post-arteriolar position and were marked green (Supplementary Table S1: https://zenodo.org/record/1229434/files/table_S1.xlsx). The exact position of the remaining capillary sheaths could not be defined. The maximal volume of a complete or incomplete sheath was about 0.0003 mm^3^ for green (Fig. 4a,b; Supplementary File S12: https://zenodo.org/record/1229434/files/File_S12.zip) and blue (Fig. 4c,d; Supplementary File S13: https://zenodo.org/record/1229434/files/File_S13.zip) sheaths.

The number of sheaths contained in each ROI was not uniform (Supplementary Table S1: https://zenodo.org/record/1229434/files/table_S1.xlsx). This was partially due to the fact that variable parts of the ROIs were occupied by regions not containing sheaths, such as follicles, larger arteries and veins. The density of capillary sheaths in the red pulp tissue proper did, however, also vary. On inspection in VR, the length and diameter of individual sheaths in one and the same ROI differed substantially. In addition, the stromal sheath cells themselves varied in shape among individual sheaths ranging from rather flat to almost prismatic. They appeared to form a continuous CD271^+^ periendothelial layer, which was, however, sometimes interrupted by unstained stromal cell nuclei and/or by intervening cells. From previous investigations^4^ it was known that CD68^+^CD163^−^ macrophages and B-lymphocytes invade the sheaths, which might provide an explanation of the latter phenomenon. These interruptions sometimes led to fragmentation of sheaths in the 3D model. Weakly SMA-positive cells in the interior of the sheaths were regarded as pericytes. It could, however, not be excluded that faint reactivity for SMA was present in single stromal sheath cells.

**Figure 5:**
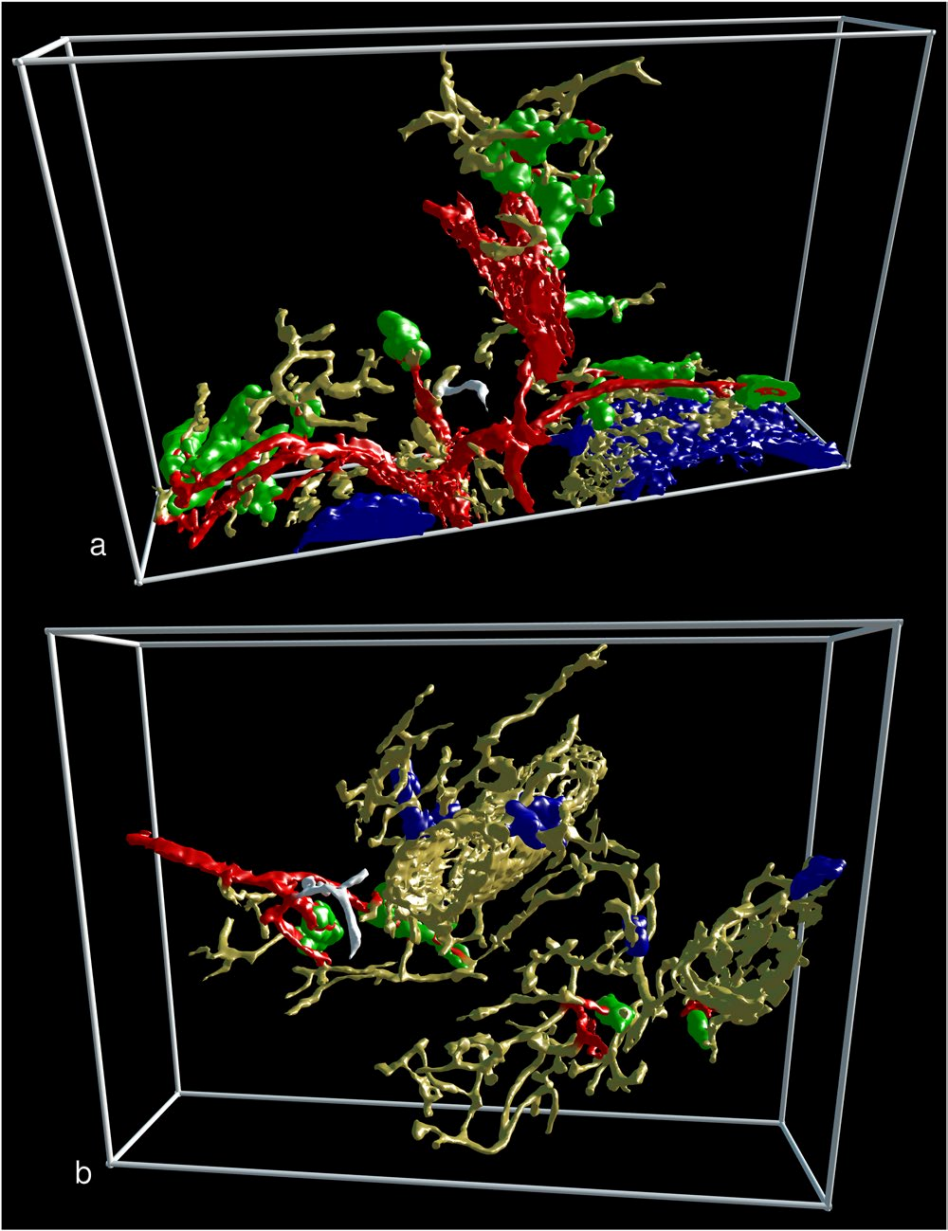
Two non-sheathed capillaries arising from an arterial vessel proximal to a capillary sheath. The non-sheathed capillaries are marked in white colour. All non-connected structures were removed. (**a**) Part of R3 seen from the last section in the series. (**b**) Part of R8 seen from the first section in the series. (**a**) and (**b**) show staining for CD34 plus SMA in yellow, for post-arteriolar CD271^+^ capillary sheaths in green and for capillary sheaths of undetectable location in blue. In (**a**) the blue structure in the lower right part corresponds to CD271^+^ FDCs in follicles. The direct connections of arterial vessels to green sheaths were manually marked in red. Length of the horizontal part of the bounding box = 882 µm in (**a**) and 709 µm in (**b**).

Several sheaths were very long and covered up to two (or even more) sequential post-arteriolar capillary branching points (Fig. 4a–d; Supplementary Files S12–13: https://zenodo.org/record/1229434), while others consisted of only a few sheath cells. Sheaths were often located distal to the branching points of arterioles (Fig. 1b). In this case, the typical wall structure of arterioles reached up to the beginning of the sheath. Thus, it was sometimes difficult to decide, whether the sheath overlapped the final part of the arteriole. However, typical smooth muscle cells were not included in sheaths for longer distances. The smaller arterial vessels often divided in a sequential dichotomous manner producing an unsheathed main vessel and a side branch with a sheath until the main vessel finally also ended in a sheath (Fig. 1b,d,f). In addition, in several ROIs we also found a single unsheathed capillary with a relatively short course and an apparently open end arising from an arteriole directly proximal to a sheath (Fig. 5a,b; Supplementary Files S14-15: https://zenodo.org/record/1229434). In such cases the sheathed capillary appeared to continue into the red pulp capillary network, while the unsheathed did not.

Thus, due to the limited number of sections, sheaths could only be identified in less than 50% of the post-arteriolar capillaries. The course of a sheathed capillary was not always straight, but sometimes involved a turn so that the sheath ran back into the direction of the feeding arteriole, as observed in supplementary file S1 (https://zenodo.org/record/1229434). Sheathed capillaries often occurred at the surface of follicles originating from arterioles bending around the follicle (Figs 1c–f and 5a). In all ROIs some sheaths even seemed to cover initial parts of the red pulp capillary network, preventing the direction of blood flow to be unequivocally recognised.

## Discussion

Our findings are based on inspection of selected regions of splenic tissue visualised as 3D models derived from serial sections stained for SMA, CD34 and CD271 using VR. This method does not only permit the observer to totally immerse into the model itself but also to inspect whether the model truly represents each individual immunostained section. Following all sections across the model permits stringent quality control (Fig. 3; Supplementary Video S4: https://zenodo.org/record/1229434/files/Video_S4.mov; Supplementary Files S1–11: https://zenodo.org/record/1229434). Inspecting a microvascular network in VR decisively helps to avoid errors due to superpositioning of structures. Such superpositioning is extremely difficult to circumvent, when a 3D model is visualised on a conventional (2D) computer screen, even if the visualisation is interactive.

We confirm that a large number of the sheathed capillaries in human spleens are located in an immediate post-arteriolar position. This may even be true for all capillary sheaths, but our series of 24 sections is too short to diagnose the location of all capillary sheaths with respect to the arterial tree. Sheathed capillaries tend to occur distal to side-branches of small arteries so that they are located close to one another (Fig. 1a,b). We highlighted all sheaths in green colour which were unequivocally connected to arterioles and arteries. Because of the limited number of sections investigated, many capillary sheaths were incomplete. Thus, in 55,5% of all sheaths detected the relation to arterioles could not be analysed, because they were only partially preserved or because the supplying vessel could not be unequivocally diagnosed.

Our models show that the length of capillary sheaths is extremely variable and that their shape is much more complicated than shown in present textbooks. A large number of sheaths continues for more than 150 µm (the estimated total length of 24 serial 7 µm sections) and may cover two or more successive capillary branching points. Sheaths of maximal volume do, however, continue outside the section series, so that their real volume remains undetermined. The size and shape of the CD271^+^ stromal sheath cells is also variable among the sheaths ranging from large isoprismatic to rather flat cells.

We have previously published the cellular composition of human splenic capillary sheaths^4,5^. They consist of endothelia with pericytes, stromal CD271^+^ sheath cells, special macrophages and recirculating CD20^+^CD27^−^ B-lymphocytes. T-lymphocytes are also found in the sheaths, but their frequency does not differ from that in the surrounding red pulp.

Our study now extends these findings by visualising splenic capillary sheaths in three dimensions. We stained both endothelia and smooth muscle cells in brown, which poses some problems in defining the exact location of the ends of arterioles, i.e., the termination of SMA staining. Most sheathed capillaries can be clearly classified as capillaries lacking smooth muscle cells. It is, however, difficult to recognize whether the stromal sheath cells sometimes initially overlap with some terminal smooth muscle cells of arterioles. In addition, it cannot be excluded that single stromal sheath cells are weakly positive for SMA. The next step of the study thus needs to involve at least three to four colours to more precisely differentiate cells associated with capillary sheaths from smooth muscle cells.

The distribution of CD271 in human spleens described by us corresponds to the description of others^24^. CD271 and SMA are present in multiple cell types. This fact necessitated a careful choice of the iso-value for mesh construction especially with respect to blue colour. CD271 also occurs in FDCs, FRCs of T-cell zones and in ubiquitous perivascular fibroblasts in the red pulp. The latter two cell populations could be excluded from the models due to their low staining intensity, but FDCs were inevitably co-visualised. The iso-values needed to be adapted for each individual ROI (Supplementary Table S2: https://zenodo.org/record/1229434/files/table_S2.xlsx). The detection of blue was chosen with the aim of excluding unwanted cell types while minimising the loss of capillary sheath staining. Losing weakly CD271^+^ capillary sheath cells and even entire sheaths could, however, not be totally avoided.

Besides smooth muscle cells, marginal reticular cells, periarteriolar fibroblasts and trabecular cells are always strongly positive for SMA. This staining could not be eliminated thus excluding automatic highlighting of arteries and arterioles. In contrast to the automatic detection of all capillary sheaths, arterial vessels supplying sheathed capillaries needed to be manually annotated, which may be error-prone.

Capillary sheaths have been described in fish^18–20^, birds^6–11,14^, pigs^13^, cats^12^, dogs^12^, humans^1–3^ and many other species of vertebrates. In animals, particulate materials and immune complexes injected intravenously initially accumulate in capillary sheaths as described for birds and fish^7,14–20^. Although not always mentioned in detail, it is likely, that this accumulation occurs in capillary sheath macrophages and not in stromal sheath cells. We suppose that the materials approach the sheaths from the outside in those species with an open splenic circulation and that they are not transported across the walls of sheathed capillaries.

Human capillary sheaths are probably involved in the initial part of naive B-lymphocyte recirculation through the spleen. Recirculating B-lymphocytes may arrive via the open circulation of the splenic cords and be then attracted to capillary sheaths. There are two reasons for this assumption: First, the B-lymphocyte accumulations around human capillary sheaths resemble a much reduced version of a special B-cell region around capillary sheaths in avian spleens called the “periellipsoid B-lymphocyte sheath”^16^. Second, we were able to detect a decisive B-cell attracting chemokine, CXCL13, in and around human stromal sheath cells^4,5^.

Interestingly, not only CD271, but also CXCL13, is described to be associated with FDCs^25^. Strong expression of both molecules may thus be a hallmark of stromal cells interacting with B-cells. Nevertheless, human sheath-associated macrophages may also be involved. These macrophages exhibit a special phenotype, which differs from that of the majority of red pulp macrophages by the absence of CD163^4^. In the vicinity of follicles, but not in the entire red pulp, human sheath-associated macrophages resemble rat marginal zone macrophages (MZMs), rat marginal metallophilic macrophages (MMMs) and mouse MMMs, because they strongly express CD169^26^. MZMs have been shown to intimately interact with B-lymphocytes^27,28^. Thus, not only stromal sheath cells, but also macrophages may attract B-lymphocytes and additionally offer contact to antigens. Signals from stromal sheath cells may directly or indirectly induce recirculating B-cells to enter the white pulp and to migrate along SMA^+^CXCL13^+/−^ periarteriolar and perarterial stromal cells heading for the surface of T-cell zones and for the mantle zones of follicles.

CD271 represents a member of the TNF receptor family with affinity for different neurotrophins and their precursors. Its function in lymphatic stromal cells has so far not been investigated. Thus, one needs to speculate why cells derived from the mesenchyme such as FDCs and, potentially, stromal sheath cells strongly express CD271. CD271 is known to be present in normal human mesenchymal and other types of stem cells and in cancer stem cells^29^. In addition, it is widely expressed in normal neurons. *In vitro*, CD271 either enhances longevity or induces apoptosis in different cell lines^29^. Recently, it has been described as an antagonist of p53^30^. Thus, CD271 may enhance the differentiation and the subsequent longevity of certain normal cell types, if strong expression is preserved. This function may be necessary, if stromal sheath cells were indispensable to initate B-lymphocyte immigration into the splenic white pulp and to channel B-lymphocytes into subsequent interactions with T helper cells.

The highly variable shape of capillary sheaths may indicate that they are dynamic structures. Sheath macrophages appear to be able to leave the sheaths^26^, if immune reactions occur. In this context, investigations on capillary sheaths in pathological human spleens are needed. Macrophages may also be of primary importance to induce perivascular fibroblasts to assume the special shape of stromal sheath cells and to become attractive for B-lymphocytes. Thus, macrophages have been described to produce neurotrophins^31^, which may interact with CD271. Macrophages themselves may not only be attracted by stromal sheath cells, but also by splenic postarteriolar endothelial cells. Our 3D-models suggest that a few post-arteriolar capillaries without sheaths also exist and that these vessels have open ends. Larger numbers of serial sections are needed to find out whether the occurrence and the shape of capillary sheaths and their specialised stromal cells depend on their exact location in the vascular tree. Microvascular blood pressure or blood flow may be a decisive variable in this context explaining why some capillaries exist without sheaths.

Why rodents and lagomorphs apparently get along without capillary sheaths in their spleens and thus form a phylogenetic exception, while the majority of vertebrates possess these structures, remains enigmatic. A convincing answer cannot be given as long as a comprehensive immunohistological review of splenic microanatomy in different species is lacking. Jeurissen *et al*.^7^ put forward the hypothesis that avian periellipsoid B-cell sheaths and their surrounding macrophages are the equivalents of rat and mouse splenic marginal zones. The cell composition of capillary sheaths does not entirely support such a hypothesis with respect to humans. Human sheath-associated B-lymphocytes phenotypically resemble naive recirculating B-cells and thus differ from the majority of rat or mouse marginal zone B-lymphocytes. The phenotype of most sheath-associated macrophages is also slightly different from rodent MZMs or MMMs.

In summary, sheathed capillaries appear to form a decisive splenic compartment which has been preserved in most vertebrate phyla including humans. This compartment merits a more detailed investigation with respect to its involvement in B-lymphocyte recirculation and stimulation.

## Methods

### Specimen

The spleen investigated came from a 22-year-old healthy male. The spleen was divided into small specimens which were fixed in 3.7% formaldehyde for 24 h and embedded in paraffin. The material was obtained in the year 2000 after the attending surgeon had informed the patient and received verbal consent that it could be used for anatomical research by the first author. This procedure corresponded to the standard in use at Marburg University Hospital at that time and was retrospectively approved by the ethics committee of the medical faculty of Marburg University.

### Immunohistology

After removal of paraffin, the sections were incubated with glucose oxidase (Sigma, St Louis, MO, No. G-6641) at 100 U/ml in PBS, pH 7.2, containing 20 mM beta-D-glucose and 2 mM NaN_3_ for 1 h at 37 °C to remove endogenous peroxidase activity. In the first step, monoclonal antibody (mAb) QBend10 (Dianova, Hamburg, Germany, No. DLN-09135) against CD34 was applied overnight at a concentration of 1:3000 in PBS/1% BSA/0.1% NaN_3_ together with mAb asm-1 (Progen, Heidelberg, Germany, No. 61001) against SMA at 1:1000. Binding of both reagents was revealed in brown colour using the Vectastain Elite system (Vector Labs, No. PK-6100 and BA-9200) for mouse IgG with diaminobenzidine as chromogen. Subsequently, the sections were autoclaved in citrate buffer pH 6.0 and mAb EP1039Y (GeneTex, No. GTX61425, via Biozol, Eching, Germany) against CD271 was used at 1: 80 overnight and revealed in blue using UltraVision for mouse and rabbit IgG (Lab Vision, Fremont, USA, via Thermo Fisher Life Technologies, Dreieich, Germany, No. TL-060-AL) and Fast Blue as chromogen. The sections were then coverslipped in polyvinyl alcohol (Mowiol 40–88, Sigma/Aldrich/Merck, No. 324590, with 9.6 g Mowiol dissolved in 48 ml glycerol/water and diluted in 48 ml Tris-HCl pH 8.5) and sealed with Eukitt.

### Digital image processing

After staining and coverslipping, the 24 serial sections were scanned using a Leica SCN 400 scanning microscope for transmitted light and a ×20 lens. The final resolution was 0.5 µm/pixel. The average thickness of a single section was measured to be 7 µm.

The acquired sections (with resolution 18.5 k × 19.5 k pixels) were initially registered using our coarse registration method^32^. After selection of regions of interest (ROIs), a fine-grain registration^33^ was applied. Each ROI had 2.5 k × 2.5 k pixel during the registration. These registered series were the input for further processing and quality control. After some initial experiments, we decided against colour deconvolution for the separation of both staining colours. Instead, we selected in CMYK (cyan-magenta-yellow-black colour space) channel C for the violet-blue chromogen and channel K for brown. The single-channel images were separately processed further. We applied inter-slice interpolation^34^ in order to reduce the 14:1 anisotropy of the data. Our interpolation method is based on dense optical flow^35^. We created intermediate images between slices to obtain a resolution of 1 µm/image in the direction of the z axis axis after interpolating the resulting volume and cropping the corresponding reference images to 2 k × 2 k pixels in the xy plane (equivalent to 1 × 1 mm). The cropping operation removed possible border effects from registration and interpolation. These operations were performed in a custom written software, which heavily utilised the Open CV library^36^. For format conversions ImageMagick (https://www.imagemagick.org) and Fiji^37^ packages were also used.

The resulting volume was further processed using 3D Slicer^38^. The typical outline was a grayscale closing operation and a Gaussian blur. The cyan channel was additionally dilated by a 11-11-3 kernel and partially exposed to a larger closing operation kernel of up to 14-14-4. The reason for this was the large colour heterogeneity in capillary sheaths, provoked by the unstained nuclei of stromal sheath cells and by unstained intercalated macrophages in the sheath. Without our processing, the reconstructed sheaths were much more inhomogeneous. In the black channel the closing operation had a radius of 7-7-2. This value was derived from other 3D reconstructions of blood vessels in human specimens. In both cases a blur with sigma value 1.0 was applied afterwards.

The present experiment was designed as a double-staining study. Thus, CD34 and SMA were both stained in brown colour. The SMA staining was primarily intended to differentiate arterioles from capillaries. However, as published previously^22,39^, SMA is also present in marginal reticular cells and in periarteriolar fibroblasts of the human splenic white pulp. In addition, it occurs in some interstitial fibroblasts in the red pulp as well as in cells of the splenic capsule and trabeculae. Thus, the reconstruction of brown-stained cells not only revealed smooth muscle cells, but also produced large fibroblast networks around arterioles and showed trabeculae. This peculiarity necessitated manual labelling of all vessels at the arterial side of the capillary sheaths in red colour.

The surface models (the meshes) were constructed using the marching cubes algorithm^40^, implemented in 3D Slicer. The typical iso-values, which determine inclusion or exclusion of a voxel with respect to a 3D surface, were about 120 (of 255) for the brown channel and about 30 for the blue channel. The actual values varied from ROI to ROI because of slightly varying staining intensity due to the different quality of fixation at the surface and in the interior of the specimen. This led to slightly variable diameters of the reconstructed capillaries in different ROIs. All iso-values are summarised in Supplementary Table S2: https://zenodo.org/record/1229434/files/table_S2.xlsx.

After mesh construction, multiple processing steps were applied. Generally speaking, the meshes were healed^41^, smoothed, and small unconnected components were removed. These operations varied based on which channel was processed. Arteriole annotations necessitated further treatment. All mesh processing except healing was performed in MeshLab^42^ (version 2016.12). We used Taubin smoothing^43^, because it does not change the size of blood vessels in the mesh representation. The processing parameters were: octree depth 9 for mesh healing and 10 iterations (the default) of Taubin smooth. In the blue channel (i.e., CD271^+^) meshes, non-connected components smaller than 5% of their main diagonal (i.e., 71 µm) were removed. In the black channel (i.e., CD34^+^ and/or SMA^+^), this was only 2%, i.e., 28 µm.

3D reconstructions from histological serial sections inevitably require quality control (QC), because multiple cell types may be stained by a single mAb and because digital processing features multiple parameters, most prominently the iso value. Wrong choice of these parameters impacts the reconstruction quality. Further, reconstructions always reveal interesting or unexpected findings that need to be more thoroughly investigated. The best way of doing so is to re-inspect the original immunohistological section in question. However, finding the correct section and communicating with the 3D reconstruction expert may be difficult. We have thus developed a custom software for QC of our reconstructions in VR. Using commodity graphics hardware (NVidia GTX 1070) with an available head-mounted display and controllers (HTC Vive), we were able to immerse the histology expert into VR. We presented the reconstructed meshes (with free choice of displayed combination) and the original data as a correctly positioned section in the virtual space (Fig. 3; Supplementary Video S4: https://zenodo.org/record/1229434/files/Video_S4.mov; Supplementary Files S1–11, https://zenodo.org/record/1229434). Thus, the user could freely move around in and through the model to investigate the original data at own choice.

The capillary sheaths were annotated by the expert immersed in VR according to their location as post-arteriolar (coloured green) or undefined (blue). Most of the sheaths in the latter category were incomplete sheaths cut at the surfaces of the ROI. The blood supply to these sheathed capillaries could not be traced, because the feeding arteriole (if present at all) was either located outside the ROI or was too short to be diagnosed. Feeding arterioles were defined by their SMA^+^ smooth muscle cells and by a characteristic arrangement of peri-arteriolar SMA^+^ fibroblasts. Both cell types and the endothelium inside the sheaths were manually labelled in red colour. At the surface of larger arteries, the majority of periarterial SMA^+^ fibroblasts remained unlabeled to more clearly visualise the course of the vessel. Each sheath which was contacted by red cells was labelled green. Thus, green sheaths were either completely included in the ROI or were cut at one of its surfaces.

We developed a special VR application for annotating arterial vessels as an interactive task. Basically, on user input, the mesh surface inside a user-controlled sphere of a given radius was coloured red. This method was implemented as a geodesic distance computation from the centre of the sphere. Thus, unintended spreading of red colour to connected or unconnected blood vessels was avoided.

The videos were encoded with FFmpeg (version 3.4.2, https://ffmpeg.org). Mesh statistics were computed with pymesh^44^.

## Data Availability

All supplementary information is to be found at https://zenodo.org/record/1229434.
